# Biocompatibilities and biodegradation of poly(3-hydroxybutyrate-*co*-3-hydroxyvalerate)s produced by a model metabolic reaction-based system

**DOI:** 10.1186/s12866-014-0285-4

**Published:** 2014-12-14

**Authors:** Suchada Chanprateep Napathorn

**Affiliations:** Department of Microbiology, Faculty of Science, Chulalongkorn University, Phayathai Road, Patumwan, Bangkok 10330 Thailand

**Keywords:** Biocompatibility, Biodegradable polymer, Polyhydroxyalkanoates, Random copolymer, Block copolymer, PHBV, Biodegradation, Cytokines, Cytotoxicity

## Abstract

**Background:**

This study evaluated the biocompatibilities of random and putative block poly(3-hydroxybutyrate*-co-*3-hydroxyvalerate)s (PHBVs) produced by a metabolic reaction-based system. The produced PHBVs were fractionated, and the copolymer sequence distributions were analyzed using ^1^H and ^13^C NMR spectroscopy. The thermal properties were analyzed using differential scanning calorimetry (DSC). Mechanical tests were conducted using a universal testing machine. The *in vitro* cytotoxicities of films composed of random PHBVs and putative block PHBVs were investigated against three types of mammalian cells. The surfaces of the copolymer films and the morphologies of the cells were qualitatively monitored using scanning electron microscopy (SEM) and atomic force microscopy (AFM).

**Results:**

Films composed of poly(3-hydroxybutyrate) (PHB), random PHBVs, putative block PHBVs, polystyrene and polyvinylchloride were prepared and characterized. The diad and triad sequence distributions indicated that the PHBVs produced via the fed-batch cultivation using two different feed systems resulted in two types of copolymers: random PHBVs and putative block PHBVs. The monomer compositions and sequence distributions strongly affected the thermal and mechanical properties. The mechanical integrity and characteristics of the film surfaces changed with the HV content. Notably, the random PHBVs possessed different mechanical properties than the putative block PHBVs. The biocompatibilities of these films were evaluated *in vitro* against three types of mammalian cells: L292 mouse connective tissue, human dermal fibroblast and Saos-2 human osteosarcoma cells. None of the PHBV films exhibited cytotoxic responses to the three types of mammalian cells. Erosion of the PHA film surfaces was observed by scanning electron microscopy and atomic force microscopy. The production of transforming growth factor-β-1 and interleukin-8 was also examined with regards to the usefulness of PHB and PHBV as biomaterials for regenerative tissue. The production of IL-8, which is induced by PHB and PHBVs, may be used to improve and enhance the wound-healing process because of deficiencies of IL-8 in the wound area, particularly in problematic wounds.

**Conclusion:**

Taken together, the results support the use of PHB and the random and putative block PHBVs produced in this study as potential biomaterials in tissue engineering applications for connective tissue, bone and dermal fibroblast reconstruction.

## Background

Advances in materials science and technology have led to rapid developments in regenerative medicine, tissue engineering and smart medical devices. Medical devices produced using synthetic polymers have been successfully applied in tissue engineering, serving as extracellular matrices to support cell growth, attachment and proliferation during *in vitro* cultivation and subsequent implantation [1]. However, the response of tissues to implanted medical devices composed of synthetic polymers can cause several adverse effects; for example, cell adhesion on untreated polymers is insufficient, the hydrophobic polymer surface prevents cell in-growth, and the lack of functional residues complicates chemical modification. Furthermore, the acidic residues of products degraded *in vivo* often induce inflammation, which has negative effects on successful applications [2].

The primary objective of tissue engineering is to reconstruct tissues and organs. Thus, implantable materials must provide mechanical support and must be compatible with cell behavior by promoting cell adhesion, proliferation and organization for the formation of functional tissue [3]. Over the past decade, polyhydroxyalkanoates (PHAs), a family of polyesters produced by microorganisms that notably include poly(3-hydroxybutyrate) (PHB), copolymers of poly(3-hydroxybutyrate-*co*-3-hydroxyvalerate) (PHBV), poly(4-hydroxybutyrate) (P4HB), copolymers of poly(3-hydroxybutyrate-*co*-4-hydroxybutyrate) (PHB-4HB), copolymers of poly(3-hydroxybutyrate-*co*-3-hydroxyhexanoate) (PHBHHx), poly(3-hydroxyoctanoate) (PHO) and their composites, have been widely applied to develop medical devices such as sutures, slings, cardiovascular patches, orthopedic pins, adhesion barriers, stents, tissue repair/regeneration devices, articular cartilage repair devices, nerve guides, tendon repair devices, bone marrow scaffolds, and wound dressings [4-8]. By manipulating the copolymer compositions, a wide range of favorable mechanical properties and control over the degradation times under specific physiological conditions can be obtained.

Various *in vitro* and *in vivo* tests have demonstrated that biomaterials composed of PHA are compatible with bone, cartilage tissue, blood and various cell lines [9-13]. Moreover, the results of *in vivo* studies have demonstrated that these materials possess various degrees of biocompatibility and biodegradability with various cell lines, collagen and stem cells [5,8,9,14-17]. Thus, the properties of PHA are being highlighted for applications in both conventional polymers and biomedical materials. However, as thoroughly reviewed by Chen and Wu [4], all studies concerning the biocompatibility of PHA as a biomaterial involved evaluating the surface properties, which must satisfy requirements for the growth of different tissues and cells. The fundamental objective of tissue engineering is to repair or replace damaged organs or tissues by delivering functional cells, supporting scaffolds, growth-promoting molecules (i.e., cytokines and growth factors) and electric or physiologic signals to areas in need [18]. A number of studies have reported that biomaterials used for tissue engineering applications should be able to elicit specific responses from the cell and thereby direct cell attachment, proliferation, differentiation, and extracellular matrix production and organization [19,20]. In particular, several studies involving the wound-healing process have reported that bioactiveinterleukin-8 (IL-8) is expressed in wounds and enhances the wound-healing process. Both growth factors (i.e., transforming growth factor-beta-1 (TGF-β-1) and IL-8) have important roles in enhancing the healing of wounds from burn injuries [21-23].

The objective of this study was to evaluate the biocompatibilities of random and putative block PHBVs produced by a model metabolic reaction-based system, which was successfully developed in a previous study [24]. In the previous study, a model predictive control (MPC)-based system consisting of two controller units was successfully developed. The first MPC unit is for on-line monitoring and for controlling the concentrations of two types of alcohols, ethanol and *n*-pentanol, and the second MPC unit is the metabolic reaction (MR) controller for automated control over the 3HV composition in PHBV [25]. The MR controller was constructed based on the metabolic flux distribution analysis of PHBV biosynthesis in *Paracoccus denitrificans* ATCC 17741 [24]. Because of the performance of this developed system, PHBVs can be produced with arbitrary 3HV contents ranging from 0 to 90 mol%. It was observed that the appearance of PHBV films produced using the optimized method differed from that of PHBV films produced using the conventional method. Next, the crystallization kinetics were evaluated under non-isothermal conditions using differential scanning calorimetry (DSC). The results indicated that PHBVs produced using the optimized method crystallized through a heterogeneous nucleation and three-dimensional growth process, whereas the PHBVs produced using the conventional method crystallized through a one-dimensional growth process [26].

This study focused on the biocompatibilities of random PHBVs and of putative block PHBVs produced by the model metabolic reaction-based system. The copolymers obtained from different processing batches were fractionated. The defined fractions were characterized based on their sequence distributions determined through ^1^H and ^13^C NMR analyses according to a previous report [27]. The physical and mechanical properties of these two types of copolymers were also investigated using DSC and a universal testing machine. To explore the potential of PHAs as biomaterials, the *in vitro* cytotoxicities of films composed of random PHBVs and putative block PHBVs toward L292 mouse connective tissue, human dermal fibroblast and Saos-2 human osteosarcoma cells were investigated. The biocompatibilities of the biomaterials were determined by monitoring the cell behavior during the adhesion of cells to their surfaces using SEM and AFM. In this study, cell growth, adhesion, proliferation and morphology and mitochondrial function were analyzed. Additionally, quantitative analyses of TGF-β-1 and IL-8 were conducted. There have been no previous reports regarding the production of TGF-β-1 and IL-8 by the cell lines being tested with PHA. To determine the usefulness of PHB and PHBV as biomaterials for the regeneration of tissue in problematic wounds due to the lack of IL-8, quantitative analyses of TGF-β-1 and IL-8 are necessary. Based on the obtained results, the potential application of PHB, random PHBVs and putative block PHBVs produced by a metabolic reaction-based system as biomaterials is discussed in terms of their properties.

## Results

### ^1^H and ^13^C NMR analyses of PHBV films

PHB, random PHBVs and putative block PHBVs were biosynthesized by *P. denitrificans* ATTC 17741 using two different feeding methods, as mentioned above. However, note that the PHBVs produced in this study were obtained using different fed-batch cultivations, as reported previously [25]. Next, the produced PHBVs were compositionally fractionated using a solution of chloroform and *n*-heptane, and the defined fractions were characterized using ^1^H and ^13^C NMR [27].

**Figure 1:**
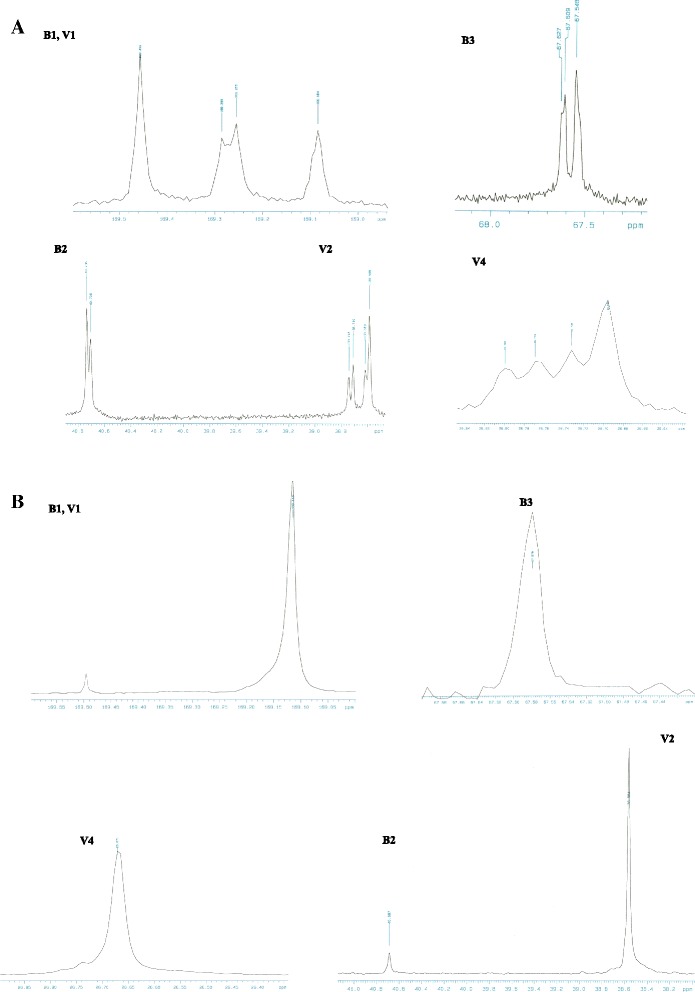
**The 600 MHz** ^**13**^ **C NMR spectra of the B1, V1, B2, B3, V2, and V4 carbon resonances of (A) random PHBV with 53 mol% of 3HV (R53) and (B) putative block PHBV with 50 mol% of 3HV (B50).**

This experiment focused on comparing the diad and triad sequence distributions between the random and putative block PHBV samples. The diad and triad sequence distributions were determined from the 600 MHz ^13^C NMR spectra, which are shown in Figure 1. The obtained relative peak intensities were interpreted in terms of the copolymer sequence distributions. The expanded CO signals of the 3HB unit (B) and of the 3HV unit (V) at 169 to 170 ppm were split into four peaks corresponding to diad sequences of B✳B, B✳V + V✳B, and V✳V. It is clear that the V-centered triad sequences of the random PHBV sample (Figure 1A) had a predominantly random sequence character. The diad and triad sequence distributions of the putative block PHBV samples that were produced using the optimized method and that were well fractionated (Figure 1B) did not show the multiple signals of B✳V + V✳B, and only the V✳V and B✳B signals were detected as a separated signal. Thus, the copolymer sequence distributions of the putative block PHBV samples are different from those of the random PHBVs. It has been reported that the ^13^C NMR spectra of random and putative block PHBV samples could exhibit a difference around 169–170 ppm and that they are sensitive to the influence of each carbonyl group of every component [28]. Next, the conditional probability for the copolymerization reaction of random PHBV and putative PHBV samples was determined by the nonlinear least squares method using the first-order Markovian model. It has been reported that this model can be applied to analyze random, block and alternative copolymerizations [27,29,30]. The probability derived from the first-order Markovian statistics and the D values of all samples are listed in Table 1. For the completely random PHBV samples, the calculated D values are close to one. For the putative block PHBV samples, the calculated D values are considerably greater than one, and their observed ^13^C NMR spectra are distinctly different from those of typical random PHBVs.

**Table 1:** **The mechanical properties of films composed of PHB, random and putative block-PHBVs, commercialized PHB, PS and PVC**

**Sample**	**3HV mol%**	**D value**	**Elongation (%) at max load**	**Stress at max load (MPa)**	**Young’s modulus (MPa)**	**Toughness (MPa)**
PHB (Sigma-Aldrich Corp.)			1.99	18.21	1068.05	1.22
PHB			1.57	20.96	1541.95	0.09
*Random PHBVs*						
*R*5	5	121	1.59	19.19	1243.94	1.77
*R*14	14	1.66	3.90	11.80	498.10	2.44
*R*23	23	1.15	13.35	10.76	331.50	3.54
*R*53	53	1.92	0.80	4.07	356.00	3.50
*R*60	60	2.11	1.70	5.91	307.44	3.22
*R*72	72	1.84	1.60	8.47	478.85	2.69
*R*80	80	1.23	2.94	11.69	507.64	2.54
*Putative block PHBVs*						
*B*12	12	14.81	1.08	1.10	78.09	0.16
*B*19	19	10.26	1.22	0.99	76.76	0.68
*B*41	41	7.92	3.64	12.05	388.14	1.41
B50	50	5.35	6.22	8.64	296.12	5.76
Polystyrene			0.36	0.99	76.76	0.57
Polyvinylchloride			1.59	120.5	386.07	2.59

### Mechanical properties

Table 1 presents the copolymer compositions and mechanical properties of the PHB homopolymer and of the random and putative block PHBVs, which were prepared as film sheets and tested under the same conditions. For comparison, commercial PHB, pure PS and PVC were also tested under the same conditions. Note that none of the films produced in this study contain nucleating agents or plasticizers. It was observed that the mechanical properties of the random PHBVs differ from those of the putative block PHBVs. The highest values were observed for the PHB, which exhibited a Young’s modulus of1541.95 MPa and a stress at maximum load of 20.96 MPa. For the random PHBVs consisting of 5%, 14%, 23%, 53%, 60%, 72% and 80% HV, the stress at maximum load and the Young’s modulus decreased as the molar fraction of 3HV increased from 0 to 60 mol%, demonstrating that the random PHBVs consisting of 0–60 mol% 3HV were more flexible and tougher. The elongation required for breaking increased as the 3HV content increased from 0 to 23 mol%. However, the random PHBVs consisting of 72 mol% and 80 mol% HV became stiff and brittle, similar to the P(3HB). These results are consistent with those in the literature [17,31]. Notably, this is the first report on the mechanical properties of putative block PHBVs consisting of 12% to 50% 3HV compared with random PHBVs consisting of 5% to 72% 3HV produced by *P. denitrificans*. The % elongation required for breaking and the toughness of the putative block PHBVs increased as the 3HV content increased. It was observed that the putative block PHBVs consisting of 12% and 19% 3HV exhibited lower % elongation, toughness, Young’s modulus, elongation and stress at maximum load than those of random PHBVs containing similar 3HV contents. However, the putative block PHBVs with HV contents of 41% and 50% possessed higher % elongation, toughness, Young’s modulus and stress at maximum load than those of random PHBVs with similar 3HV contents. Compared with pure PS and PVC prepared using the same procedures, PHB and random PHB with 5% 3HV exhibited similar % elongations, but their toughness was lower than that of PVC. The block PHBV with 50% 3HV exhibited the highest toughness and % elongation among the polymers investigated in this study.

### Cytotoxicity evaluation

**Figure 2:**
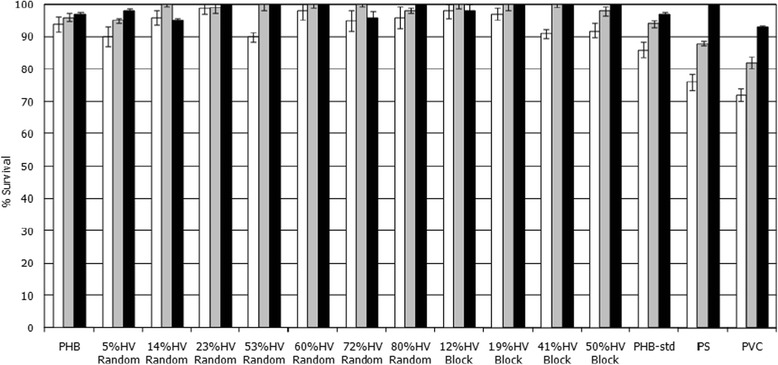
**The cytotoxicity results of L292 mouse connective tissue, human dermal fibroblast and Saos-2 human osteosarcoma cells when separately grown on films composed of PHB, random and putative block PHBVs, commercial PHB, PS and PVC.** White bars, L929 mouse connective tissue; grey bars, Saos-2 human osteosarcoma; black bars, human dermal fibroblast.

The cytotoxicities of PHA film sheets composed of PHB and of random and putative block PHBVs were evaluated against three types of mammalian cell lines: L292 mouse connective tissue, Saos-2 human osteosarcoma cells and human dermal fibroblasts. The percent viabilities of the cells were compared to those of the cells cultured in 12-well plates and treated only with fresh medium (control experiment). The MTT conversion data are presented in Figure 2.

All three cell lines grew well on all of the produced PHA film sheets. In this study, the percent viability increased over time in all samples, including the control. However, the L292 mouse connective tissue and human dermal fibroblasts clearly proliferated at significantly higher rates on the produced PHA film sheets than on the chemically synthesized PS and PVC film sheets. There was no significant difference between the random and putative block PHBVs with respect to cell proliferation. Both appeared to be non-toxic to all three types of cell lines and exhibited percent viabilities that were better than those of the PS and PVC film sheets.

### Biocompatibility evaluation

**Figure 3:**
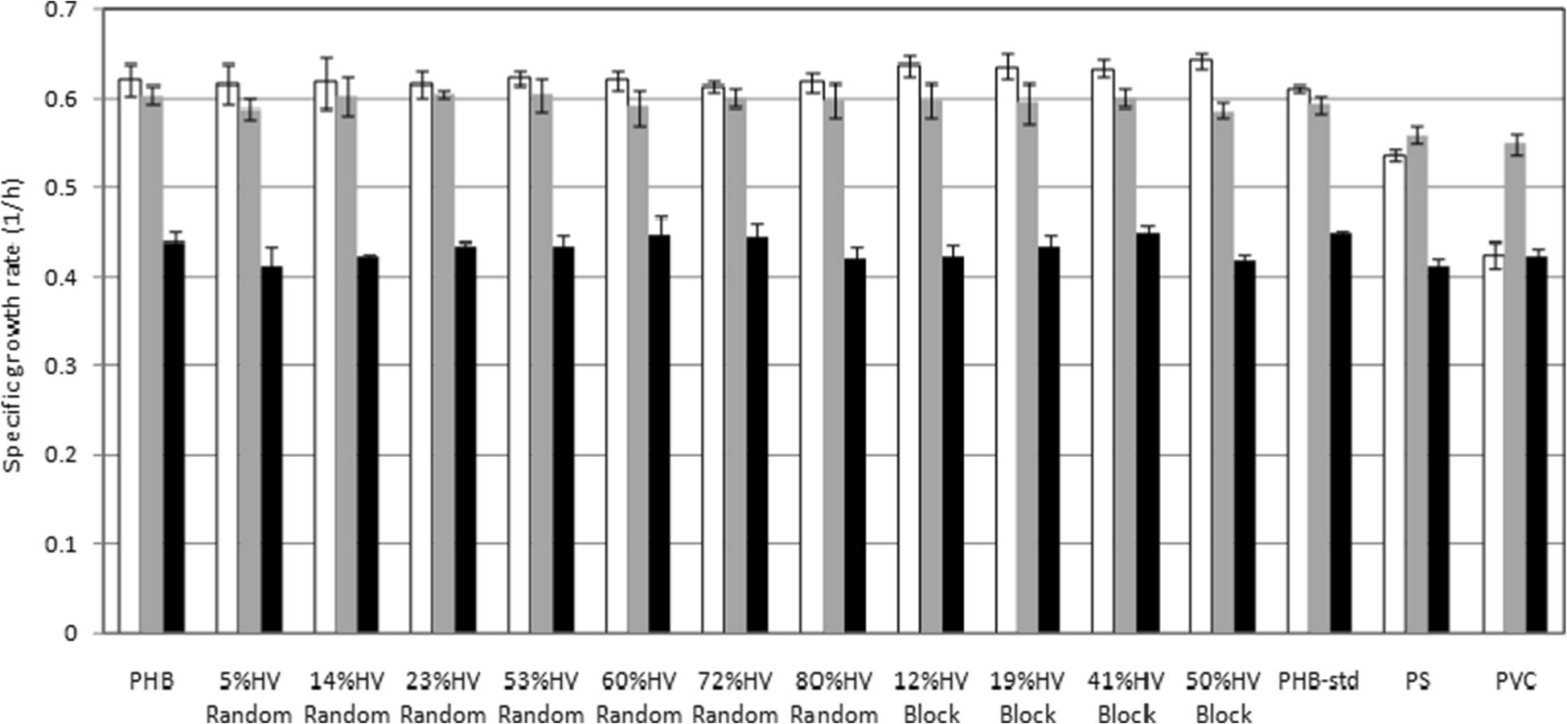
**The specific growth rates of L292 mouse connective tissue, human dermal fibroblast and Saos-2 human osteosarcoma cells when separately grown on films composed of PHB, random and putative block PHBVs, commercial PHB, PS and PVC for 7 days.** White bars, L929 mouse connective tissue; grey bars, Saos-2 human osteosarcoma; black bars, human dermal fibroblast.

To evaluate the biocompatibility for the attachment and proliferation of cells on the surfaces of the PHA film sheets, each cell type was cultured on the surfaces of the films for 7 days. The proliferation of the cells was determined every 24 hours for 7 days. Figure 3 presents the specific growth rates of L292 mouse connective tissue, Saos-2 human osteosarcoma cells and human dermal fibroblasts on PHA film sheets after the cells were cultured for 7 days. The cell densities and cell line viabilities increased until confluency on day 3 for both L292 mouse connective tissue and Saos-2 human osteosarcoma cells and on day 5 for dermal fibroblasts, whose cell density and viability subsequently declined until day 7. It was observed that human dermal fibroblasts exhibited a specific growth rate that was slower than those of L292 mouse connective tissue and Saos-2human osteosarcoma cells. The effects of the monomer composition and the difference in the random and block sequence distributions on the specific growth rates of all three cell lines were not observed in this study.

### Production of TGF-β-1 and IL-8

**Figure 4:**
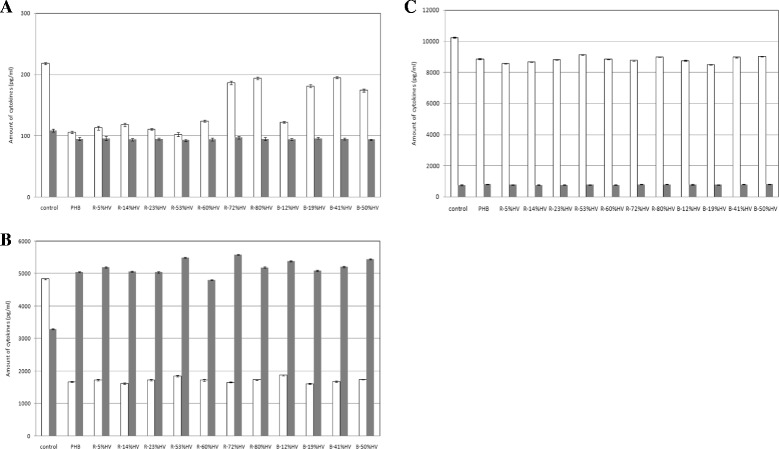
**The production of TGF-β-1 and IL-8 from (A) L292 mouse connective tissue, (B) human dermal fibroblast and (C) Saos-2 human osteosarcoma cells when grown separately on films composed of PHB and random and putative block PHBVs for 7 days.** White bars, TGF-β-1; grey bars, IL-8.

TGF-β-1 is a growth factor that is produced by various types of cells. The amount of TGF-β-1 produced by cells is very low under normal conditions. Many studies have reported the involvement of TGF-β-1 and IL-8 during wound healing, inflammation, remodeling and bone formation [32]. In this study, L929 mouse connective tissue cells, human dermal fibroblast cells and Saos-2 human osteosarcoma cells were cultured on the surfaces of the PHB and random PHBV and putative block PHBV film sheets. The amounts of TGF-β-1 and IL-8 secreted into the cultured supernatant were determined using ELISA test kits. The control experiments were performed in the same way, except PHB or PHBV film sheets were not placed at the bottom of each well. As shown in Figure 4, 102–195, 1622–1875 and 8483–9118 pg/mLTGF-β-1 were produced from L292 mouse connective tissue (Figure 4A, white bars), human dermal fibroblasts (Figure 4B, white bars) and Saos-2 human osteosarcoma cells (Figure 4C, white bars), respectively, when grown on PHA film sheets for 7 days. However, there appears to be an obvious decrease in TGF-β-1 levels compared with their respective control levels, as shown in Figures 4A,B and C. Interestingly, opposite trends were observed when comparing the production of TGF-β-1 and IL-8 from human dermal fibroblasts (Figure 4B) and from Saos-2 human osteosarcoma cells (Figure 4C). Saos-2 osteosarcoma cells grown on PHA film sheets produced more TGF-β-1 than IL-8, whereas human dermal fibroblasts grown on PHA film sheets produced more IL-8 than TGF-β-1. Notably, as shown in Figure 4B, human dermal fibroblasts produced significantly more IL-8 than did the controls, but they produced less TGF-β-1 than did the controls. In Figure 4A, the effect of the monomeric composition in PHBV shows thatL929 mouse connective tissue cells grown on random 72%HV, random 80%HV, putative block 19%HV, putative block 41%HV and putative block 51%HV appear to have almost 2-fold higher levels of TGF-β-1 than L292 cells grown on PHB and random PHBV with low 3HVcontents. The effect of the monomeric composition in putative block PHBVs appears to be higher than that observed in random PHBVs with similar 3HV contents. However, the levels of TGF-β-1 produced from all three types of mammalian cells did not exceed the control levels.

### SEM and AFM analyses of the surfaces of PHBVs with and without cell attachment

**Figure 5:**
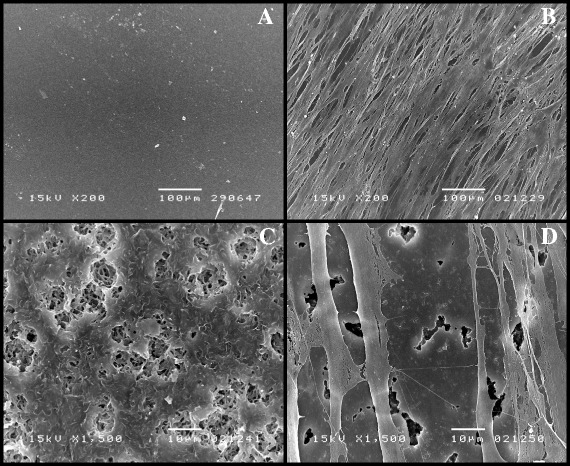
**The SEM images of human dermal fibroblasts cultured on a film sheet composed of random PHBV with 23% 3HV content; the surface of the film sheet (A) before cell cultivation, bar 100 μm; (B) after cell cultivation for one month, bar 100 μm; (C) the opposite side of the same film sheet after cell cultivation for one month, bar 10 μm; (D) the same film with human dermal fibroblast cells grown on the surface for one month, bar 10 μm.**

To assess the microstructures and morphologies of the film surfaces, SEM and AFM images of cells grown on PHB and on random and block PHBV film sheets were regularly recorded over one month. Figure 5 presents typical scanning electron micrographs (SEMs) of the surface of a random PHBV with 23% 3HV. The original surface of the random PHBV with 23% 3HV (Figure 5A) was compared with the film surface after culturing human fibroblast cells on the film sheets for one month (Figure 5B,C and D). As shown in Figure 5B, the human dermal fibroblast cells could grow and proliferate very well on the random PHBVs. The cells exhibited the normal and healthy characteristics of human dermal fibroblast cells. Figure 5C shows the opposite side of the same film sheet. After culturing for one month, the film surface ultimately eroded; human dermal fibroblast cells were directly attached to the observed erosion sites (Figure 5D).

**Figure 6:**
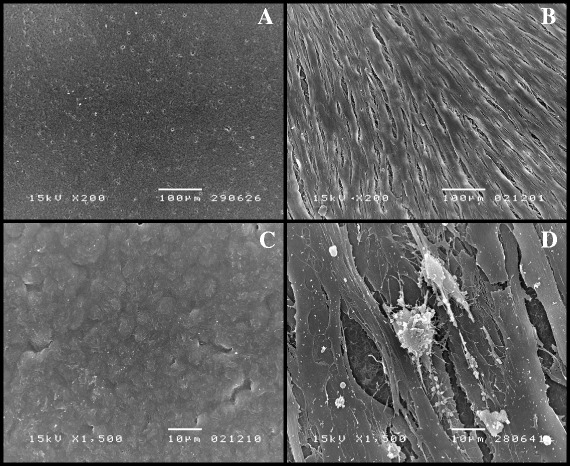
**The SEM images of human dermal fibroblasts cultured on a film sheet composed of putative block PHBV with 19% 3HV content; the surface of the film sheet (A) before cell cultivation, bar 100 μm; (B) after cell cultivation for one month, bar 100 μm; (C) the opposite side of the same film sheet after cell cultivation for one month, bar 10 μm; (D) the same film with human dermal fibroblast cells grown on the surface for one month, bar 10 μm.**

Figure 6 presents the SEMs of surfaces of putative block PHBVs with 19% 3HV under the same conditions. Figure 5A shows the original film sheet, and Figures 6B,C and D show human dermal fibroblast cells and the surface of the film sheet after cell culturing for one month. The human dermal fibroblasts grew very well on the putative block PHBVs (Figure 6B). A small erosion of the film surface of putative block PHBVs was also observed, but this erosion was less than that on the random PHBVs.

**Figure 7:**
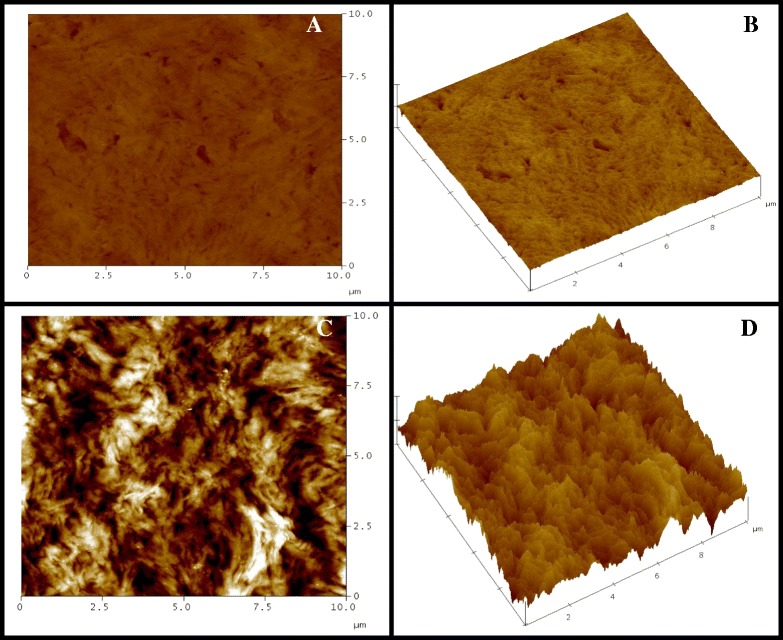
**The AFM images of human dermal fibroblasts cultured on a film sheet composed of random PHBV with 23% 3HV content; (A) 2-D image and (B) 3-D image of the original surface of the film sheet before cell cultivation for one month; (C) 2-D image and (D) 3-D image of the same film sheet after cell cultivation for one month.**

**Figure 8:**
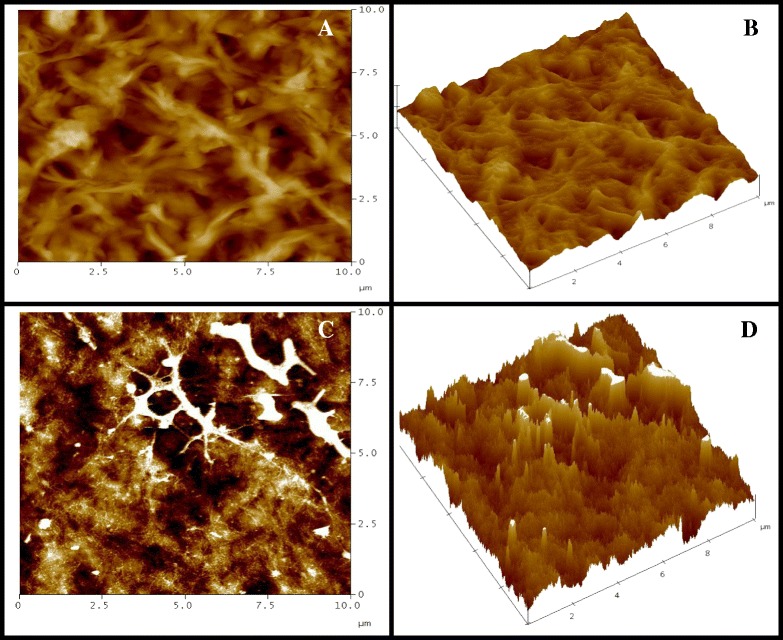
**The AFM images of human dermal fibroblasts cultured on a film sheet composed of putative block PHBV with 19% 3HV content; (A) 2-D image and (B) 3-D image of the original surface of the film sheet before cell cultivation for one month; (C) 2-D image and (D) 3-D image of the same film sheet after cell cultivation for one month.**

To evaluate these changes, tapping mode AFM measurements were conducted using a scanning probe microscope(SPM), the NanoScope. The results are shown in Figures 7 and 8. Figure 7A and B show the original surface of random PHBVs with 23% 3HV. The film possesses a smooth surface. However, after culturing human dermal fibroblast cells on the surfaces of the film sheets for one month, as shown in Figure 7C and D, similar erosions of the film surface were observed as that in the SEM analysis. Figure 8A and B show the original surface of block PHBV with 19% 3HV. The film surface of the block PHBV was rich with crystalline regions and rougher than the random PHBV surface with a similar 3HV content. Film surface erosion was not observed even after culturing human dermal fibroblast cells on the surface of film sheets for one month, as shown in Figure 8C and D. The cells attached on the surface are shown in white. Similar observations were made for L292 mouse connective tissue and Saos-2 human osteosarcoma cells.

## Discussion

Previous studies have shown that homopolymers of PHB and random and putative block PHBVs can be successfully produced with arbitrary 3HV contents from 0 to 80 mol% using an automated feed strategy based on a metabolic reaction system. It was found that the different feeding strategies of mixed carbon sources, ethanol and *n*-pentanol, strongly affected the thermal properties of the PHBV copolymer [26], enzyme degradation rates [27] and, in this study, mechanical properties. The random and putative block PHBVs exhibited different mechanical characteristics due to their different monomeric sequence distributions (Figure 1). The enzyme degradation rates of random and putative block PHBVs were different because the block PHBV was rich in long-sequences of block 3HB units but less rich in 3HV random regions [27]. The kinetics of the non-isothermal crystallization of block PHBV was faster than that of the random PHBV due to the frequent occurrence of block 3HB units [26]. Therefore, the difference in monomeric sequence distribution affects the thermal properties, biodegradable properties and mechanical properties [33]. Because the surface microstructure and monomeric sequence distribution may also affect the biocompatible properties, we investigated the biocompatibilities of the produced PHB and random and putative block PHBVs *in vitro* against three different mammalian cell lines.

First, the cytotoxicities of PHB and random and putative block PHBVs with 3HV contents from 0-80% were investigated using MTT assays (Figures 2 and 3). The MTT assays and the cell growth kinetics revealed that PHB and random and putative block PHBVs showed evidence of non-toxicity toward L292 mouse connective tissue, human dermal fibroblast and Saos-2human osteosarcoma cells, leading to increased proliferation in terms of increased cell viabilities and specific growth rates [34-36]. However, decreases in cell viability and delays in cell growth were observed in PS and PVC. This finding can be explained based on the fact that monomeric 3HBA is an intermediate that naturally occurs in human blood [14,37]. The surface properties of these PHBV samples may promote cell attachment and proliferation by providing sufficient gas and nutrient exchange or more serum protein adsorption. In addition, PHBVs may possess adequate hydrophobic surface areas to induce the selective adsorption of cells because of the natural hydrophobic character of cells [38]. In this study, the effects of the monomeric sequence distribution between random and block copolymers on cell viability and growth were not observed.

Growth factors and cytokines are considered to be therapeutic candidates because they are synthesized by and stimulate cells required for tissue repair (e.g., platelets, macrophages, endothelial cells, keratinocytes and fibroblasts). Growth factors and cytokines are deficient in chronic wounds, and pharmacological applications on wounds enhance the wound repair process. The wound-healing process normally occurs in three stages: (1) the directed and sequential migration of neutrophils, monocytes, keratinocytes and fibroblasts into the wound during the first several days; (2) the activation of wound macrophages and fibroblasts, resulting in the *de novo* synthesis of growth factors, cytokines and extracellular matrix proteins and the proliferation of fibroblasts in the successive 2–3 weeks; and (3) remodeling with active collagen turnover and cross linking from two weeks to one year [39,40]. Therefore, this study focused on the influences of PHB and PHBV on the production of TGF and IL-8 by human dermal fibroblasts (Figure 4). It was found that L292 mouse connective tissue and Saos-2 human osteosarcoma cells, when grown on PHB or random or putative block PHBVs, did not produce significant amounts of TGF and IL-8 compared with the control experiments. However, human dermal fibroblasts secreted significant amounts of IL-8 that were much higher than in the control experiment. It is known that fibroblasts secrete a broad array of cytokines that are closely involved in the wound-healing process, including TGF-β-1 and IL-8. There has been little research regarding the effects of biomaterials on the production of TGF-β-1 and IL-8 by human dermal fibroblast cells, except for the work by Lv et al. [41], who reported the systematic effects of chitosan on fibroblasts derived from hypertrophic scars and keloids. Three types of fibroblasts, hypertrophic scar fibroblasts, keloids fibroblasts and normal dermal fibroblasts, were cultured in the presence or absence of indicated dosages of chitosan, ranging from 20 to 320 μg/ml, for 72 hours. These authors reported that treatment with chitosan could increase the production of IL-8 but dramatically reduce the production of TGF-β-1, both in a dose-dependent manner. They concluded that chitosan regulated the production of TGF-β-1 and IL-8, providing more evidence that chitosan has the potential to be used as wound-healing and tissue repair agent or as a dressing material [41].

In this study, PHB and random and putative block PHBVs may contribute factors that induce human dermal fibroblast cells to produce IL-8. With regards to the usefulness of PHB and PHBV as biomaterials for the regeneration of tissue, the production of IL-8,which is induced by PHB and PHBVs, may be used to improve and enhance the wound-healing process because of deficiencies of IL-8 in the wound area, particularly in problematic wounds such as burn infections and decubitus ulcer problems and in secondary wounds from systemic diseases.

The morphologic evaluation (Figures 5, 6, 7 and 8) revealed that human dermal fibroblast cells grew well on PHB and random and block PHBV matrices, which AFM confirmed by revealing the three-dimensional growth of cells on the matrix. Combined with the results shown in Figure 3, the specific growth rates of L929 mouse connective tissue, Saos-2 human osteosarcoma and human dermal fibroblast cells grown on PS were 0.53 ± 0.01, 0.56 ± 0.01 and 0.41 ± 0.01 1/h, respectively. The specific growth rates of all three types of cell lines grown on standard PHB, produced PHB, random PHBVs and putative block PHBVs were higher than those on PS. In contrast, PVC resulted in lower specific growth rates for all three types of cell lines compared to PS. The produced PHB and random and putative block PHBVs were cell-supportive materials based on the appearance of spindle-shaped cells, which are attained only after proper cell adhesion to a substrate. The SEM and AFM results clearly showed the ability of human dermal fibroblast cells to adhere and proliferate on random and block PHBV surfaces. At the same time, cell cultures of different origins, including L292 mouse connective tissue and Saos-2 human osteosarcoma cells, in direct contact with PHB and random and putative block PHBVs films exhibited high levels of cell adhesion [9,37,42,43]. Indeed, the erosion rates of random and block PHBV film surfaces were similar to those reported in a previous study performed using *in vitro* enzymatic degradation with PHA depolymerase from *P. lemoignei*. By comparison, the degradation rates of block PHBV film sheets were slower than those of random PHBV film sheets for similar ranges of mol% 3HV, indicating that the degradation rates of these two polyesters were dependent on the 3HV content and the monomeric sequence distribution [27].

The results indicate that all of the PHA films could be applied as biomaterials to support cell growth and cell attachment for all three cell lines. Finally, taken together with the cell proliferation, density and morphology of mammalian cells grown on these biodegradable film sheets, these results demonstrate the potential for using PHAs as biomaterials due to their ability to support mammalian cell growth.

## Conclusions

The PHB and the random and putative block PHBVs produced by the fed-batch cultivation of *P. denitrificans* with a feeding strategy based on a model metabolic reaction-based system are biocompatible with three mammalian cell types and induce human dermal fibroblasts to secrete IL-8,one of the cytokines required for the wound-healing process. Taken together, the results support the use of the random and putative block PHBVs produced in this study as candidates for biomaterials in tissue engineering for connective tissue, bone and dermal fibroblast reconstruction.

## Methods

### Synthesis of random and putative block PHBVs

PHB homopolymer and random and block PHBV copolymers were synthesized by *P. denitrificans* ATTC 17741 from ethanol and *n*-pentanol using two different feeding methods. These two methods were described in previous reports [24,25].

### Polymer purification and fractionation

The dried cells were packed in filter paper (Whatman 1002–042, Sigma-Aldrich Corp., St. Louis, MO, USA) and refluxed in hot chloroform in a Soxhlet apparatus to separate PHBV from the dried cells. The PHA was recovered from the chloroform by precipitation in *n*-hexane. The precipitation step was repeated three times. The PHBV samples were compositionally fractionated with a chloroform and *n*-heptane solution [26].

### Analysis of chemical composition distribution in the polymers by ^1^H and ^13^C NMR

Proton (^1^H) and carbon (^13^C) NMR spectra were recorded at 30°C in CDCl_3_ on a Varian NMR spectrometer (Varian Inova 600 MHz; Palo Alto, CA, USA). The 3HV contents in the P(HB-*co*-HV) samples were determined from the relative intensities of the methyl resonance of the 3HV and 3HB units in the ^1^H NMR spectra. The chemical composition distributions of the samples were determined by analyzing the carbonyl resonance in the ^13^C NMR spectra. The D value was defined for evaluating the selectivity of the copolymerization reactions as *D* = *P*_ii_*P*_jj_/(*P*_*ij*_*P*_ji_), where *P*_*ij*_ indicates the mole fraction of the B✳V diad sequence (*i* = B and *j* = V). The conditional probability for the copolymerization reaction of the V component to the B-terminal of the polymer chain was estimated by a nonlinear least squares method using the first-order Markovian model based on the ^1^H and ^13^C NMR data, in which fractions of diad and triad sequence intensities were involved [27].

### Preparation of PHA films for mechanical tests

The PHA films used for the mechanical properties test were prepared according to ASTM: D882-91 (American Society for Testing and Materials, D882-91) [44]. The PHA films were prepared using conventional solvent-casting techniques from chloroform solutions of polyesters with glass trays (Pyrex, Corning Incorporated, NY, USA) as the casting surface [27]. The thicknesses of the polyester thin films were regulated by controlling the concentration of the polymer in chloroform at 1% (w/v) and the volume of the polymer solution. The thickness of the PHA films was 0.05 mm, which was confirmed using an Absolute Digimatic Caliper (Model 500–175: CD-12″C, Mitutoyo Corporation, Kawasaki-shi, Kanagawa, Japan). A minimum of fifteen 50 × 150 mm film samples were cut and aged for one month to reach equilibrium crystallization prior to the analyses.

### Mechanical properties test

Mechanical tests were conducted using a Universal Testing Machine (Lloyd LRX, Lloyd Instruments Ltd., Fareham Hampshire, UK) with a crosshead speed of 10 mm/min. The measured variables included the elongation at break (%), stress at maximum load (MPa), toughness (MPa) and Young’s modulus (MPa). Commercial PHB (Sigma-Aldrich Corp., St. Louis, MO, USA), pure polystyrene (PS) and pure polyvinylchloride (PVC) (Wako Pure Chemical Industries, Ltd., Osaka, Japan) were also tested under the same conditions for comparison. The data represent the mean value of fifteen samples tested under the same conditions.

### Preparation of PHA films for biocompatibility tests

Films of produced PHB, block PHBV, random PHBV, commercial PHB, PS and PVC were prepared using solvent-casting techniques, as described above. The average PHA film thickness was regulated by controlling the concentration of PHA in chloroform at 2.5% (w/v). The thicknesses of the film samples were measured using an Absolute Digimatic Caliper (Model 500–175: CD-12″C, Mitutoyo Corporation, Kawasaki-shi, Kanagawa, Japan), and the film thickness was approximately 0.1 mm. The films were cut to 10 × 10 mm and aged for one month to reach equilibrium crystallization, and their initial weights were measured prior to analysis. The PHA film sheets were sterilized by immersion in 70% ethanol for 30 minutes, followed by drying sterilization under UV irradiation for 60 minutes.

### Cell culture

Mouse connective tissue cells (L292: ECACC Cat. No. 85011425) and human dermal fibroblast cells isolated from normal human foreskin tissue were cultured in Dulbecco’s Modified Eagle’s Medium (DMEM) supplemented with 10% fetal bovine serum, 2 mM L-glutamine, 100 units/mL penicillin and 100 μg/mL streptomycin. Human osteosarcoma cells (Saos-2: ATCC Cat. No. HTB-85) were cultured in alpha Minimum Essential Medium (MEM) supplemented with 10% fetal bovine serum, 2 mM L-glutamine, 0.1 mM MEM non-essential amino acid, 1.0 mM sodium pyruvate, 100 units/mL penicillin and 100 μg/mL streptomycin. The cells were incubated at 37°C in a fully humidified atmosphere of 5% CO_2_ in air. The proliferative cells in the log phase were detached using 0.25% trypsin and 0.02% EDTA and were counted using a hemocytometer. The optimal cell density was evaluated, which revealed that a density of 1 × 10^4^ cells/well (12-well tissue culture plate, Nunc A/S, Roskilde, Denmark) was optimum.

### Cytotoxicity evaluation based on microtitration test (MTT) assay

Evaluation of the indirect cytotoxicities of the PHA films was performed in accordance with published standard methods (BS-EN30993-5 and ISO10993-5) [45]. Cell suspensions of L292 mouse connective tissue, human dermal fibroblast and Saos-2 human osteosarcoma cells were separately seeded at a density of 1.0 × 10^4^ cells/well in 12-well plates (Nunc A/S, Roskilde, Denmark) that contained a PHA film sheet at the bottom of each well. After 24 hours of exposure, the medium was removed from the cell cultures, and the cells were re-incubated for an additional 24 hours in fresh medium and then evaluated using the MTT assay. Briefly, 50 μL of (3-(4,5-dimethylthiazol-2-yl)-2,5-diphenyltetrazolium bromide) (MTT 5 mg/mL in PBS) and 450 μL of serum-free DMEM were added to each well followed by 4 hours of incubation at 37°C to allow for the formation of formazan crystals. Subsequently, the media were replaced with 200 μL of dimethyl sulfoxide (DMSO) and 25 μL of Sorensen’s glycine buffer, pH10.5, and incubated for an additional 30 minutes. The absorbance at 570 nm was measured using a microplate reader (Molecular Devices LLC, Sunnyvale, CA, USA). The data were analyzed using the SoftMax Program (Molecular Devices LLC, Sunnyvale, CA, USA) to determine the percent viability of cells when cultured with extraction media in comparison with the control experiment. The data are representative of three independent experiments and are the mean values ± the standard deviation (SD) of the independent experiments.

### Biocompatibility evaluation for the attachment and proliferation of cells

Each PHA film sheet was placed in a 12-well tissue culture plate (Nunc A/S, Roskilde, Denmark). Next, 100-μL single cell suspensions containing 1 × 10^5^ cells were seeded on the surfaces of the PHA film sheets in each well and were allowed to attach at 37°C for 15 minutes prior to the addition of 900 μL of fresh culture medium. The proliferation of the cells on each sheet was determined by washing the cells twice with PBS to remove non-adherent cells, and the numbers of attached and viable cells, including non-adherent cells suspended in culture medium, were determined every 24 hours for 7 days. To examine cell growth, the culture medium was replaced after 24 hours with fresh culture medium. The cell density of cells attached to the polymer and the total cell density were determined. The data are representative of three independent experiments and are the mean values ± SD of the independent experiments.

### Production of TGF-β-1 and IL-8

To evaluate the effects of the cell-biomaterial interactions on the production of TGF-β-1 and IL-8, the amounts of TGF-β-1 and IL-8 secreted into the cultured supernatant were determined using ELISA test kits obtained from R&D Systems according to the manufacturer’s instructions. The production of TGF-β-1 and IL-8 by the cells cultured with or without PHA films was evaluated by collecting the culture medium, which was changed every 24 hours for up to 7 days. The data are representative of three independent experiments and are the mean values ± SD of the independent experiments.

### The cell morphology and distribution and PHA film surface morphologies with and without attached cells analyzed by SEM and AFM tapping mode measurements

After 1, 3, 5 and 7 days of growth, the PHA films with cells attached to their surfaces were washed twice with PBS and then immersed in PBS containing 2% glutaraldehyde (pH 7.4) for 2 hours at 4°C. The samples were dehydrated using a graded ethanol series (from 30%, 50%, 70%, 90% to 100% ethanol). The samples were then mounted on aluminum stubs and coated with gold using an ion coater. The surfaces of the PHA films and the morphologies of the attached cells were observed using a scanning electron microscope (JSM-5410 LV; JEOL, Tokyo, Japan). AFM was used to qualitatively monitor the surfaces of the PHA films and the morphologies of cells grown on the PHA films over a scale range of 100 nm to 10 μm. AFM tapping mode measurements were conducted using a SPM (NanoScope IV; Veeco, Santa Barbara, CA, USA) with a nanoscope controller (Version 5.30r3sr3, Veeco, Santa Barbara, CA, USA) at room temperature. The cantilever used in this study was a silicon microtip with a spring constant of 20 N/m. The AFM technique allows for high-resolution imaging of the surface structures of biomaterials and cells without labeling.
